# Glucose metabolic disorder in Klinefelter syndrome: a retrospective analysis in a single Chinese hospital and literature review

**DOI:** 10.1186/s12902-021-00893-5

**Published:** 2021-12-01

**Authors:** Shixuan Liu, Tao Yuan, Shuoning Song, Shi Chen, Linjie Wang, Yong Fu, Yingyue Dong, Yan Tang, Weigang Zhao

**Affiliations:** 1 Department of Endocrinology, Key Laboratory of Endocrinology of National Health Commission, Peking Union Medical College Hospital, Peking Union Medical College & Chinese Academy of Medical Sciences, No. 1 Shuaifuyuan Street, Dongcheng District, Beijing, 0086 100730, China; 2 Department of Pharmacy, Peking Union Medical College Hospital, Peking Union Medical College & Chinese Academy of Medical Sciences, No.1 Shuaifuyuan Street, Dongcheng District, Beijing, 0086 100730 China

**Keywords:** Klinefelter syndrome, Diabetes mellitus, Hyperglycemia, Insulin resistance, Hypoglycemic therapy

## Abstract

**Background:**

We aimed to investigate the clinical characteristics and islet β-cell function in patients with Klinefelter syndrome (KS) and hyperglycemia.

**Methods:**

This is a retrospective study. In total, 22 patients diagnosed with KS were identified from the electronic medical record system, including 9 patients with hyperglycemia (total patients with hyperglycemia, THG-KS group) and 5 hyperglycemic KS patients with oral glucose tolerance test (OGTT) results (HG-KS group). An additional 5 subjects with hyperglycemia and 5 normal glucose tolerance (NGT) subjects matched based on body mass index were included as the HG group and NGT group, respectively. Data from clinical and laboratory examinations were collected. We further performed a literature review of KS and hyperglycemia.

**Results:**

We found that KS patients developed abnormal glucose metabolism earlier in life than those without KS, and the median age was 17 years, ranging from 10 years to 19 years. Six of 17 (35.3%) patients were diagnosed with diabetes mellitus and 3 of 17 (17.6%) patients were diagnosed with prediabetes. Among 10 patients with both fasting blood glucose and insulin results recorded, there were 8 out of 17 (47.1%) KS patients had insulin resistance. The prevalence of hypertension and dyslipidemia was higher in patients with hyperglycemia and KS than in patients with NGT KS. Compared with the HG group, insulin sensitivity levels were lower in HG-KS group, whereas homeostasis model assessment of β-cell function levels (*p* = 0.047) were significantly, indicating higher insulin secretion levels in the HG-KS group.

**Conclusions:**

KS patients develop hyperglycemia earlier in life than those without KS and show lower insulin sensitivity and higher insulin secretion. These patients also have a higher prevalence of other metabolic diseases and may have different frequencies of developing KS-related symptoms.

**Supplementary Information:**

The online version contains supplementary material available at 10.1186/s12902-021-00893-5.

## Background

Klinefelter syndrome (KS) is the most frequent sex chromosome disorder of the male population [1], and the estimated prevalence of KS ranges from 1 in 500 to 1 in 1000 in males [2]. KS is characterized by hypergonadotropic hypogonadism with decreased levels of androgens and inhibin B, which cause increased secretion of follicle stimulating hormone (FSH) and luteinizing hormone (LH) through a negative feedback loop [3] and other classical phenotypes, including aspermatogenesis [4], tall and slender bodies with narrow shoulders, long arms and legs, small testes and sparse body hair [5]. The genetic background of KS involves the presence of one or more extra X chromosomes, and the most universal karyotype is 47 XXY, accounting for approximately 80–90% of patients [1]. Other karyotypes, including 47 XXY/46 XY chimaeras, 48 XXXY, 48 XXYY or 49 XXXXY, have also been detected in the remaining KS patients [6].

Previous studies have observed that KS is associated with the development of diabetes mellitus (DM), insulin resistance, hyperinsulinemia, hyperlipidemia, obesity and other metabolic diseases [7]. The prevalence of overt DM in KS is estimated to be greater than 10% depending on the population [8], and abnormal oral glucose tolerance test (OGTT) results are detected in approximately greater than one-third of KS patients [9]. These subjects are characterized by an earlier onset age and a lower body mass index (BMI) than the general population [10]. Epidemiological studies have found that the morbidity and mortality of DM in KS are greater than threefold increased [11, 12]. However, most previous studies focused on either the prevalence of DM or metabolic syndrome among KS patients or different features between KS patients with and without DM, and only a few studies have studied the characteristics of islet β-cell function between hyperglycemic patients with and without KS.

In this study, we retrospectively summarized the characteristics of patients with hyperglycemia and KS from a single Chinese hospital database, compared clinical features, insulin sensitivity and islet β-cell secretion function between hyperglycemic subjects with and without KS, and further performed a literature review. We aimed to describe the clinical features and characteristics of islet β-cell function in patients with KS and hyperglycemia and to help select the appropriate hypoglycemic therapy for DM patients with KS.

## Materials and methods

### Subjects

This was a retrospective study. The electronic medical record system of Peking Union Medical College Hospital (PUMCH) was used to identify patients with a final diagnosis of KS from January 2000 to December 2019 by searching the clinical notes. KS was diagnosed according to the definite diagnosis of KS in other hospitals or karyotyping records, and 22 KS patients were identified. After excluding 5 patients without electronic records of laboratory tests, 8 KS patients had normal glucose tolerance (NGT-KS group, *n* = 8), and 9 KS patients had hyperglycemia (total patients with hyperglycemia, THG-KS group, *n* = 9). Among these 9 patients, 4 patients diagnosed with DM but without records of OGTT were excluded, and the remaining 5 patients, including 2 DM patients and 3 prediabetes patients, were enrolled in the KS and hyperglycemia group (HG-KS group, *n* = 5). All patients did not start testosterone treatment at the time of collecting the clinical data. An additional 10 subjects, including 5 subjects with hyperglycemia but without KS (HG group, *n* = 5) and 5 NGT subjects (NGT group, *n* = 5), were matched with patients in the HG-KS group based on BMI.

The diagnosis of DM was based on the diagnostic criteria of the American Diabetes Association [13]. Prediabetes was defined as fasting blood glucose (FBG) between 6.1 mmol/L and 6.9 mmol/L or glycosylated hemoglobin A1c (HbA1c) from 5.7 to 6.4% or 2-h postprandial blood glucose (PBG) during 2-h OGTT between 7.8 mmol/L and 11.1 mmol/L and no diagnosis of DM. Hyperglycemia included the states of DM and prediabetes. Obesity was defined as BMI ≥ 28 kg/m^2^ according to diagnostic criteria for the Asian population [14]. Hypertension was diagnosed based on the following criteria: systolic blood pressure (SBP) ≥130 mmHg, diastolic blood pressure (DBP) ≥85 mmHg, or the use of antihypertensive medications. Dyslipidemia was diagnosed based on the following criteria: elevated serum triglyceride (TG) (> 1.7 mmol/L), low serum high density lipoprotein cholesterol level (HDL-c) (< 1.04 mmol/L), or the use of lipid-lowering agents [15].

This study was approved by the PUMCH Ethics Committee and followed the ethical standards of the responsible committee on human experimentation (institution and national) and the Helsinki Declaration of 1964, as revised in 2013. All participants provided written consent for the inclusion of materials pertaining to themselves and acknowledged that they could not be identified via the paper. The participants were fully anonymized.

### Clinical data and oral glucose tolerance test

Clinical history, physical examination and laboratory examination results were collected from the medical database during the period of admission. BMI was calculated as weight (kg) divided by the square of the height in meters (m^2^). Blood pressure was measured thrice after 5 min of rest and was recorded as the mean value of three measurements.

Blood samples were collected at baseline (0 min), as well as 30 min, 60 min, 120 min and 180 min after 75-g anhydrous glucose load by oral administration after fasting for 8 to 12 h. Blood samples were used for serum glucose, insulin and C-peptide assays. The quantitative insulin sensitivity check index (QUICKI) [16], insulin sensitivity index proposed by Matsuda et al. (ISImatsuda) [17], the reciprocal of the product of fasting serum insulin and blood glucose referred to as the insulin action index (IAI), the ratio of the area under the curve of glucose and insulin (AUC_Glu_/AUC_Ins_) [18] and homeostasis model assessment of insulin resistance (HOMA-IR) [19] were calculated to reflect insulin resistance. In addition, homeostasis model assessment of β-cell function (HOMA-β) values [19] and area under the curve values for insulin (AUC_Ins_) were calculated to reflect islet β-cell secretion function.

### Literature review

We searched PubMed for manuscripts with full texts in English published prior to February 2020 using key words “Klinefelter syndrome” OR “Klinefelter’s syndrome” AND “diabetes mellitus” OR “DM” OR “insulin resistance” OR “hyperglycemia” OR “impaired glucose tolerance” OR “impaired fasting glucose” OR “prediabetes” OR “metabolic syndrome”. Two coauthors extracted the medical information of the enrolled patients and the literature using standardized forms independently. If any difference was noted, another coauthor assisted in checking differences between the two coauthors. Twelve studies [9, 11, 20–29] and 10 case reports [30–39] of KS combined with DM or prediabetes were selected.

### Statistical analysis

Continuous variables are expressed as the mean ± standard deviation. Student’s *t* test was used to compare differences between continuous variables of each group, and the continuous variables that failed the normality test were logarithmically transformed before analysis or tested by the nonparametric test. A *p*-value less than 0.05 was considered significant. All statistical analyses were performed using the statistical program SPSS (version 25, SPSS, Chicago, IL).

## Results

### Characteristics of our patients

Among the 17 KS patients recruited in this study, 35.3% (6 out 17) of patients were diagnosed with DM, 17.6% (3 out 17) of patients diagnosed with prediabetes, and 47.1% (8 out 17) of patients presented insulin resistance with HOMA-IR ≥ 2.5. The clinical data of KS patients in the PUMCH center are summarized in Table 1. Of the total of 10 patients presenting with recordings of karyotype results, all presented the classical 47 XXY karyotype. The prevalence of hypertension and dyslipidemia were both increased in the THG-KS group (57.1 and 85.7% for hypertension and dyslipidemia, respectively) compared with the NGT-KS group (12.5 and 40.0% for hypertension and dyslipidemia, respectively). Compared with the NGT-KS group, the prevalence of cryptorchidism (14.3% in the THG-KS group vs 60.0% in the NGT-KS group) was considerably reduced in the THG-KS group, whereas the ratio of gynecomastia was considerably increased (75.0% in the THG-KS group vs 50.0% in the NGT-KS group).

**Table 1:** Clinical data of patients with Klinefelter Syndrome in PUMCH center

Characteristics	KS (*n* = 17)	THG-KS (*n* = 9)	NGT-KS (*n* = 8)
Age (y)	18.6 ± 5.4	19.6 ± 6.9	17.5 ± 2.7
Height (cm)	176.9 ± 11.4	180.7 ± 11.4	172.5 ± 9.5
Body weight (kg)	72.2 ± 19.8	76.2 ± 19.3	67.6 ± 19.3
BMI (kg/m^2^)	22.76 ± 4.48	23.00 ± 3.77	22.49 ± 5.17
SBP (mmHg)	128.9 ± 22.4	144.7 ± 22.45	115.1 ± 13.3
DBP (mmHg)	78.5 ± 20.0	89.9 ± 21.7	68.5 ± 11.1
Testes size (ml)	2.9 ± 1.6	1.5 ± 1.5	2.4 ± 1.5
T (ng/ml)	1.84 ± 1.22	2.24 ± 1.52	1.43 ± 0.57
FSH (IU/L)	25.88 ± 16.84	23.30 ± 12.13	29.32 ± 25.71
LH (IU/L)	29.23 ± 19.72	30.37 ± 13.48	27.72 ± 25.71
TC (mmol/L)	4.95 ± 2.00	5.39 ± 2.30	4.34 ± 1.22
TG (mmol/L)	2.22 ± 1.26	2.57 ± 1.41	1.73 ± 0.77
LDL-c (mmol/L)	2.79 ± 1.09	2.94 ± 1.27	2.61 ± 0.79
HDL-c (mmol/L)	1.02 ± 0.21	1.05 ± 0.22	0.99 ± 0.19
Clinical features			
Decreased testosterone levels	14/14 (100.0%)	7/7 (100.0%)	7/7 (100.0%)
Increased gonadotropin levels	11/13 (84.6%)	4/6 (66.7%)	7/7 (100.0%)
Infertility	4, others were not considering fertility when collecting the data	4	0
Small testicles (adults)	4/7 (57.1%)	1/3 (33.3%)	3/4 (75.0%)
Decreased pubic hair (adults)	7/8 (87.5%)	3/4 (75.0%)	4/4 (100.0%)
Gynecomastia	5/11 (45.5%)	3/7 (75.0%)	2/4 (50.0%)
Behavioral and intelligence problems	2/13 (15.4%)	1/8 (12.5%)	1/5 (20.0%)
Delayed secondary sexual characteristics	10/13 (76.9%)	4/7 (57.1%)	6/6 (100.0%)
Cryptorchidism	4/12 (33.3%)	1/7 (14.3%)	3/5 (60.0%)
Obesity	4/13 (30.8%)	1/7 (14.3%)	3/6 (50.0%)
Hypertension	5/17 (29.4%)	4/7 (57.1%)	1/8 (12.5%)
Dyslipidemia	8/12 (66.7%)	6/7 (85.7%)	2/5 (40.0%)
Kayotype	10/10 (100%) 47 XXY		
Insulin resistance	8/17 (47.1%)		

Abbreviations: *PUMCH* Peking Union Medical College Hospital; *BMI* body mass index; *SBP* systolic blood pressure; *DBP* diastolic blood pressure; *T* testosterone; *FSH* follicle stimulating hormone; *LH* luteinizing hormone. *TC* total cholesterol; *TG* triglyceride; *LDL-c* low density lipoprotein cholesterol; *HDL-c* high density lipoprotein cholesterol. Insulin resistance was defined as HOMA ≥2.5. HOMA was calculated as a measure of insulin resistance as follows: [fasting blood glucose (mmol/L) × fasting insulin (μIU/mL)]/22.5

### Characteristics of islet β-cell function in KS and hyperglycemia patients

KS patients developed abnormal glucose metabolism earlier in life compared with those without KS (*p*<0.01) (Table 2). The average age of HG-KS was 16.2 ± 3.3 years old, and the median age was 17 years old, ranging from 10 to 19 years old. No significant difference in BMI was noted between subjects in the HG group and HG-KS group because we matched BMI when enrolling subjects. Between the two groups with hyperglycemia, FINS and HOMA-IR values were higher in the HG-KS group, and ISImatsuda, QUICKI, IAI and AUC_Glu_/AUC_Ins_ were lower in the HG-KS group. However, no significant differences were noted. HOMA-β (*p* = 0.047) values were significantly increased in the HG-KS group compared with those with hyperglycemia only. In addition, AUC_Ins_ were also increased in the HG-KS group, but no significant differences were noted.

**Table 2:** Characteristics of patients of hyperglycemia with or without KS and normal glucose tolerance subjects

	HG-KS (*n* = 5)	HG (*n* = 5)	NGT (*n* = 5)	*p* value	
				HG-KS vs HG	HG-KS vs NGT
Age (y)	16.2 ± 3.3	41.2 ± 2.1	34.0 ± 6.2	0.008*	0.009*
BMI (kg/m^2^)	22.68 ± 2.97	24.82 ± 2.00	22.08 ± 2.50	0.600	0.754
FBG (mmol/L)	5.90 ± 2.04	7.54 ± 3.21	5.22 ± 0.37	0.251	0.754
FINS (μIU/ml)	29.22 ± 26.00	12.66 ± 7.01	8.89 ± 2.62	0.076	0.009*
HOMA-IR	7.47 ± 7.05	5.22 ± 5.47	2.10 ± 0.75	0.175	0.032*
HOMA-β	346.24 ± 202.59	76.64 ± 28.69	103.56 ± 20.07	0.047*	0.047*
ISImatsuda	35.98 ± 13.87	61.17 ± 23.05	88.29 ± 29.67	0.076	0.009*
QUICKI	0.30 ± 0.03	0.32 ± 0.03	0.35 ± 0.02	0.175	0.028*
IAI	0.01 ± 0.005	0.02 ± 0.008	0.02 ± 0.008	0.175	0.028*
AUC_Ins_	423.89 ± 254.66	146.06 ± 62.23	179.62 ± 69.83	0.175	0.175
AUC_Glu_/AUC_Ins_	0.17 ± 0.25	0.31 ± 0.17	0.14 ± 0.05	0.117	0.175

Statistical difference is along the row and *p*<0.05 was considered significant

*represents significant difference between two groups

Abbreviations: *KS* Klinefelter syndrome, *HG* hyperglycemia, *NGT* normal glucose tolerance, *BMI* body mass index, *FBG* fasting blood glucose, *FINS* fasting serum insulin, *HOMA-IR* homeostasis model assessment of insulin resistance, *HOMA-β* homeostasis model assessment of β-cell function, *ISI*_*matsuda*_ insulin sensitivity index proposed by Matsuda et al., *QUICKI* quantitative insulin sensitivity check index, *IAI* insulin action index, *AUC*_*Ins*_ area under curve of insulin, *AUC*_*Glu*_*/AUC*_*Ins*_ ratio of area under curve of glucose and insulin

**Fig. 1:**
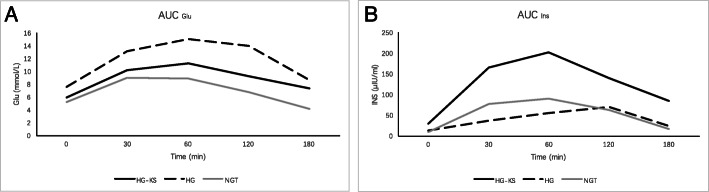
The increment curves of serum glucose (**A**) and insulin (**B**) during OGTT in HG-KS group, HG group and NGT group. Abbreviations: *OGTT* Oral glucose tolerance test, *KS* Klinefelter syndrome, *HG* hyperglycemia, *NGT* normal glucose tolerance, *AUC* area under curve, *Glu* glucose, *Ins* insulin

**Fig. 2:**
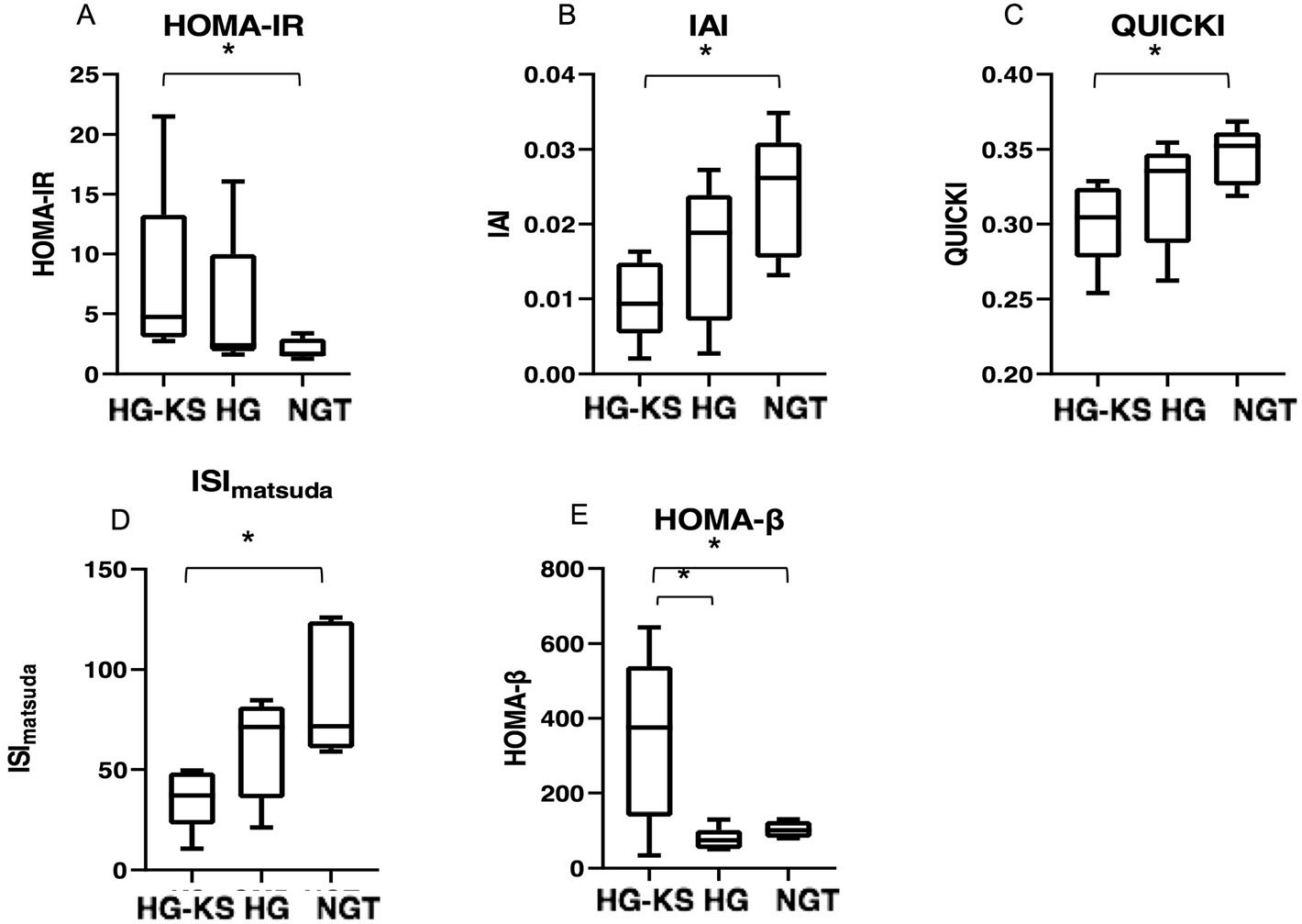
Boxplots of HOMA-IR (**A**), IAI (**B**), QUICKI (**C**), ISImatsuda (**D**) and HOMA-β (**E**) in HG-KS group, HG group and NGT group. * represented significant difference (*p*<0.05) between two groups. HOMA-IR (**A**) (*p* = 0.032) was significantly increased in HG-KS group compared to NGT group, IAI (**B**) (*p* = 0.028), QUICKI (**C**) (*p* = 0.028) and ISImatsuda (**D**) (*p* = 0.009) was significantly decreased in HG-KS group compared to NGT. HOMA-β (**E**) was significantly increased in HG-KS group compared to both HG (*p* = 0.030) and NGT (*p* = 0.044) groups. Abbreviations: *KS* Klinefelter syndrome, *HG* hyperglycemia, *NGT* normal glucose tolerance, *HOMA-IR* homeostasis model assessment of insulin resistance, *HOMA-β* homeostasis model assessment of β -cell function, *ISImatsuda* insulin sensitivity index proposed by Matsuda et al., *QUICKI* quantitative insulin sensitivity check index, *IAI* insulin action index

In the comparison between the HG-KS group and NGT group, HOMA-IR (*p* = 0.032) was significantly increased in the HG-KS group, and ISI_matsuda_ (*p* = 0.009), QUICKI (*p* = 0.028) and IAI (*p* = 0.028) were significantly decreased. HOMA-β (*p* = 0.047) levels were higher in the HG-KS group. Figure 1 shows the serum insulin and glucose increment curves based on the OGTT in the HG-KS group, HG group and NGT groups. Figure 2 shows the characteristics of insulin sensitivity and islet β-cell secretion function related parameters in these three groups.

### Literature review

In the literature review, previous studies showed that the prevalence of DM in KS patients ranged from 6.8 to 39%, and the prevalence of insulin resistance in KS patients ranged from 24.0 to 38.5% (Table 3). By summarizing the characteristics of 12 patients in previous case reports (with detailed clinical data) and 9 patients in the THG-KS group (Table 4), we found that the average onset age of hyperglycemia was 27.75 ± 11.8 years. Among 16 patients with data of sex hormone records, 15 presented hypergonadotropic hypogonadism, and the remaining patient presented decreased testosterone levels. The most common clinical feature related to KS was decreased testosterone levels (100.0%) followed by increased gonadotropin levels (93.8%), decreased pubic hair (88.9% in adults), small testicles (83.3% in adults), delayed secondary sexual characteristics (63.6%), behavioral and intelligence problems (31.3%), gynecomastia (28.6%) and cryptorchidism (20.0%). All patients with infertility plans complained of infertility. Regarding karyotypes, 71.4% patients were 47 XXY, 14.3% were 46 XY/47 XXY and 14.3% were 49 XXXXY.

**Table 3:** Literature review of studies evaluating diabetes mellitus or insulin resistance in Klinefelter syndrome

Author, year (ref.)	Number of patients	Age (year)	BMI (kg/m^2^)	DM (%)	IFG (%)	IR (%)	Diagnosed criteria of IR	Karyotype
Han, 2016 [20]	376	32	24.7 ± 3.9	28 (12.8%)	57 (26.0%)	–	–	47 XXY, 354; 48 XXXY, 2; 48 XXYY, 1; 46 XY/47 XXY, 13; 47 XXY/ 48 XXXY/ 46 XY, 3; 47 XXY/46 XY/46 XX, 1; 47 XXY/48 XXXY, 1; 47 XX, inv. (Y), 1
Yesilova, 2005 [21]	13	22	23.7 ± 4.9	–	–	38.5%	Glucose disposal rates < 4.53 mg/kg/min in hyperinsulinemic euglycemic clamp	All 47 XXY
Bojesen, 2006 [11]	70 (35 without TRT/35 with treatment)	35/39	27.3/25.1	3 (8.5%)/4 (11.4%)	6 (17.1%);7 (20.0%)	–	–	–
Falhammar, 2018 [22]	224	22	26.1 ± 5.3	9.10%	–	–	–	47 XXY, 204; 47 XXY/46 XY, 6; 47 XXY/46 XX, 3; Others and unknown, 5; 46 XX testicular males, 6.
Ota, 2002 [23]	895	43	21.5 ± 4.44	61 (6.8%)	–	–	–	47 XXY, 40; 46 XY/47 XXY, 9; 48 XXYY, 2; 47 XXY/48 XXXY/46 XY, 1; 47 XXY/46 XY/46 XX, 1; unknown, 8.
Bardsley, 2011 [29]	89 Prepubertal Boys	8	–	0	0	20 (24%)	HOMA ≥2.5	47 XXY, 84; 48 XXYY, 1; 47 XXY/46 XY, 2; 46 XX translocation, 1.
Jackson, 1966 [24]	8	–	–	–	1 (12.5%)	–	–	47 XXY, 2; others unknown.
Becker, 1966 [25]	50	38	–	5 (10.0%)	–	–	–	–
Pasquali, 2013 [26]	69	31	27.5	3	16	–	–	–
Nielsen, 1969 [9]	31	–	–	12 (39%); especially 47 XXY/46XY, 4; 47 XXY, 5; 48 XXXY, 3.	–	–	–	47 XXY/46 XY, 4; 47 XXY, 24; 48 XXXY, 3.
Davis, 2016 [27]	96 Prepubertal Boys	–	–	0	0	9 (33.3%), only 27 patients calculated for HOMA	HOMA ≥2.5	47 XXY, 88; 46 XY/47XXY, 2; 48 XXXY, 1; 48 XXYY, 1; 46 XX + SRYtrans, 1.
Davis, 2017 [28]	93 Prepubertal Boys	–	–	–	1	–	–	47 XXY, 89; 46 XY/47 XXY, 2; 48 XXXY or 48 XXYY, 3.

Abbreviations: *TRT* testosterone replacement therapy, *T* testosterone, *FSH* follicle stimulating hormone, *LH* luteinizing hormone, *BMI* body mass index, *DM* diabetes mellitus, *IFG* impaired fasting glucose, *FBG* fasting blood glucose, *HbA1c* hemoglobin A1c, *HOMA* homeostatic model assessment, *IR* insulin resistance. Continuous variables were expressed as mean or mean ± standard deviation (SD). HOMA was calculated as a measure of insulin resistance as follows: [fasting blood glucose (mmol/L) × fasting insulin (μIU/mL)]/22.5

**Table 4:** Abnormalities associated with Klinefelter syndrome and hyperglycemia combined PUMCH center and previous case reports

Characteristics	Patients (*n* = 21)
Age	27.75 ± 11.8
Clinical features	
Decreased testosterone levels	16 out 16 (100.0%)
Increased gonadotropin levels	15 out 16 (93.8%)
Infertility	7 adults with recording
Small testicles (adults)	10 out 12 (83.3%)
Decreased pubic hair (adults)	8 out 9 (88.9%)
Gynecomastia	4 out 14 (28.6%)
Behavioral and intelligence problems	5 out 16 (31.3%)
Delayed secondary sexual characteristics	7 out 11 (63.6%)
Cryptorchidism	3 out 15 (20.0%)
Obesity	9 out 18 (50.0%)
Hypertension	8 out 15 (53.3%)
Hyperglycemia	9 out 13 (69.2%)
Karyotype	10 out 14 (71.4%), 47 XXY; 2/14 (14.3%), 46 XY/47 XXY; 2 out 14 (14.3%), 49 XXXXY
Prediabetes	3 out 21 (14.3%)
Diabetes mellitus	18 out 21 (85.7%)
Insulin resistance parameters	8 out 10 (80.0%)

Abbreviations: *PUMCH* Peking Union Medical College Hospital

Insulin resistance was defined as HOMA≥2.5. HOMA was calculated as a measure of insulin resistance as follows: [fasting blood glucose (mmol/L) × fasting insulin (μIU/mL)]/22.5

The specific clinical data of patients with hyperglycemia in both the PUMCH center and previous literature are summarized in Supplementary Table 1.

## Discussion

In this study, we summarized the clinical features of KS patients in a single Chinese hospital center and evaluated the characteristics of islet β-cell function in KS and hyperglycemia patients compared with hyperglycemia patients without KS and NGT subjects. The prevalence of DM in KS patients in the PUMCH center was 35.3%. This value was much higher than the prevalence of DM in the general population, which was 10.4% in China according to the guidelines of the Chinese Diabetes Society published in 2017 and 14.3% in the United States [40]. KS is considered as a state of “prediabetes” [41]. In addition, associations between KS and impaired glucose tolerance and DM have been reported, and several possible mechanisms of DM have been proposed. Low levels of testosterone are proposed to correlate with the increased prevalence of insulin resistance and DM in males [42, 43]. In several studies in KS patients, testosterone deficiency was identified as an independent predictor for insulin resistance and metabolic syndrome [10, 20], and the effects of testosterone replacement therapy (TRT) on ameliorating hyperglycemia and insulin resistance [44] were observed. The gene dosage effect from the extra copies of X chromosomes was hypothesized to be another factor [6] based on the evidence of the close relationship between karyotypes and DM [6, 9], and the level of insulin resistance [45]. Autoimmune abnormalities may also be involved given the presence of T1DM-related autoantibodies in some KS patients [10, 46]. Other mechanisms, such as changes in body composition, inflammation status [11], socioeconomic factors [2], high triglyceride levels, fatty liver and acute pancreatitis [6], might also play important roles in the development of DM in KS patients. However, the specific pathogenesis remains to be elucidated, and further large, long-term, prospective, randomized, controlled studies are needed to clarify whether and to what extent the above factors may affect glycemic metabolism in KS patients.

We found that hyperglycemia developed earlier in life in KS patients compared with those without KS, which was consistent with previous observations that found that the course of DM in KS patients was approximately 30 years old [10]. Insulin sensitivity was lower in hyperglycemic KS patients compared with hyperglycemic patients without KS, whereas HOMA-β levels were significantly higher. These results indicated better competence of insulin compensatory secretion. Insulin resistance was considered the major characteristic in KS patients with DM. Bojesen et al. [11] calculated insulin sensitivity using the HOMA model and showed a significantly decreased insulin sensitivity but a significantly increased islet β-cell secretion function in KS patients. Pei et al. [47] observed elevated insulin resistance in KS patients using the area under the curve of serum insulin after a 75-g oral glucose load and insulin suppression test. Using the gold standard, the hyperinsulinemic euglycemic clamp test, Lee et al. [48] demonstrated that impaired peripheral insulin resistance was the underlying mechanism of impaired glucose tolerance in Korean patients with KS. In contrast, Yesilova et al. [21] discovered that plasma insulin levels of KS patients were significantly elevated, but insulin-mediated glucose disposal values were not reduced compared with the controls. These researchers concluded that hyperinsulinemia may be the primary metabolic abnormality rather than insulin resistance. Our results found that insulin resistance and a compensatory increase in insulin secretion exist in KS patients, and the increased islet β-cell secretion function was statistically significant compared with those patients with hyperglycemia but without KS.

Best practices for hypoglycemic therapy in hyperglycemic KS patients have still not been established. The effects of TRT on the improvement of glucose control remain controversial. Some clinical trials observed improvement in HbA1c levels after TRT [44, 49], whereas others reported no improvement [50–52]. In particular, improvements in insulin sensitivity by TRT were observed in obese hypogonadal patients but not lean patients [53]. According to previous evidence, populations with lower testosterone levels tend to have a higher proportion of body fat, which results in impaired insulin sensitivity. After TRT, improvement in the ratio of fat and muscle composition could benefit glucose and lipid metabolism rather than the direct effect of TRT. Insulin therapy is a common strategy in KS patients with DM. In the case review in Japan, among 895 Japanese KS patients reported in literature up to 2001, 61 patients were diagnosed with DM, and at least 20 patients were treated with insulin preparations. However, glycemic control was poor with an HbA1c level of 10.6% [23], reflecting less effective in glycemic control of insulin therapy among KS and DM patients. Based on the results of the changes in insulin sensitivity and islet β-cell secretion function in our study, we found that KS patients with hyperglycemia presented with similar insulin resistance levels but better islet β-cell secretion function than those without KS. These findings suggested that insulin preparations might not represent the best choice for KS patients with hyperinsulinemia given that hyperinsulinemia and insulin resistance would result in an increased dosage of insulin preparations and reduce the curative effects. Further weight gain following an increased insulin dosage would aggravate insulin resistance. From this point of view, oral hypoglycemic drugs that improve insulin sensitivity might be considered first for those with existing islet β-cell secretion function. For KS patients with hyperglycemia, we recommended individualized hypoglycemia drug selection after evaluating islet β-cell function rather than taking insulin therapy at first.

Based on the results of clinical features associated with KS, those patients with hyperglycemia were more likely to present gynecomastia, which was consistent with the findings of lower testosterone levels compared with those with NGT. The prevalence of cryptorchidism was lower in hyperglycemia patients, whereas the prevalence of behavioral and intelligence problems was increased. We also found an increased frequency of development of other metabolic diseases in hyperglycemia and KS patients, including hypertension and dyslipidemia. These results confirmed that other metabolic factors, including blood pressure and serum lipid levels, may have effects on glucose metabolism in KS patients.

This study has some limitations. First, KS is a rare disease, and this is a retrospective study in a single Chinese center. Thus, the sample size was small. Second, the clinical information was limited, and data on some clinical features were missing. Third, the age of patients in the HG-KS group did not match that in the HG group because adolescents with type 2 diabetes mellitus were mostly obese, thus the BMI values of these patients could not be matched with those of KS patients.

## Conclusions

In conclusion, we found that KS patients develop hyperglycemia earlier in life.

compared with those without KS. Patients with KS and hyperglycemia were more likely to have other metabolic diseases and may have different frequencies of developing KS-related symptoms than NGT KS patients. The prevalence of gynecomastia and behavioral and intelligence problems was higher, whereas the prevalence of cryptorchidism was lower. KS patients with hyperglycemia tended to have decreased insulin sensitivity and hyperinsulinemia and increased insulin secretion compared with hyperglycemia patients without KS. Based on the characteristics of glucose metabolism in KS patients, we recommend evaluating islet β-cell function before hypoglycemic treatment, given that oral hypoglycemic drugs may be the first choice for those still with islet β-cell secretion function rather than insulin preparations, because decreased insulin sensitivity may have a poor hypoglycemic effect.

## Supplementary Information

**Additional file 1.** Supplementary Table 1. Characteristics of patients in our center and previous case reports

## Data Availability

The data generated during and/or analysed during the current study are available from the corresponding author on reasonable request.

## Abbreviations

KS: Klinefelter syndrome
FSH: follicle stimulating hormone
LH: luteinizing hormone
DM: diabetes mellitus
OGTT: oral glucose tolerance test
BMI: body mass index
PUMCH: Peking Union Medical College Hospital
NGT: normal glucose tolerance
FBG: fasting blood glucose
HbA1c: glycosylated hemoglobin A1c
PBG: postprandial blood glucose
SBP: systolic blood pressure
DBP: diastolic blood pressure
TG: triglyceride
HDL-c: high density lipoprotein cholesterol level
QUICKI: Quantitative Insulin Sensitivity Check Index
ISImatsuda: insulin sensitivity index proposed by Matsuda et al.
IAI: the reciprocal of the product of fasting serum insulin and blood glucose which named insulin action index
AUC_Glu_/AUC_Ins_: the ratio of area under curve of glucose and insulin
HOMA-IR: homeostasis model assessment of insulin resistance
HOMA-β: homeostasis model assessment of β-cell function
TRT: testosterone replacement therapy
T1DM: type 1 diabetes mellitus
